# Association of Early and Supernormal Vascular Aging categories with cardiovascular disease in the Chinese population

**DOI:** 10.3389/fcvm.2022.895792

**Published:** 2022-08-11

**Authors:** Qiuyu Cao, Mian Li, Tiange Wang, Yuhong Chen, Meng Dai, Di Zhang, Yu Xu, Min Xu, Jieli Lu, Weiqing Wang, Guang Ning, Yufang Bi, Zhiyun Zhao

**Affiliations:** ^1^Department of Endocrine and Metabolic Diseases, Shanghai Institute of Endocrine and Metabolic Diseases, Ruijin Hospital, Shanghai Jiao Tong University School of Medicine, Shanghai, China; ^2^Shanghai National Clinical Research Center for Metabolic Diseases, Key Laboratory for Endocrine and Metabolic Diseases of the National Health Commission of the PR China, Shanghai Key Laboratory for Endocrine Tumor, State Key Laboratory of Medical Genomics, Ruijin Hospital, Shanghai Jiao Tong University School of Medicine, Shanghai, China

**Keywords:** aging, cardiovascular diseases, mortality, risk factors, vascular stiffness

## Abstract

**Background:**

Early Vascular Aging and Supernormal Vascular Aging are two extreme phenotypes of vascular aging, and people in the two categories demonstrate distinct clinical characteristics and cardiovascular prognosis. However, the clinical implication of vascular aging categories in the Asian or Chinese population has not been investigated. We aimed to investigate the association between vascular aging categories and cardiovascular events in a Chinese cohort.

**Methods:**

We explored the association of vascular aging categories with incident cardiovascular disease in a community cohort in Shanghai, China, which included 10,375 participants following up for 4.5 years. Vascular age was predicted by a multivariable linear regression model including classical risk factors and brachial-ankle pulse wave velocity. Early and Supernormal vascular aging groups were defined by 10% and 90% percentiles of Δ-age, which was calculated as chronological minus vascular age.

**Results:**

We found that cardiovascular risk significantly increased in Early [hazard ratio (HR), 1.597 (95% CI, 1.043–2.445)] and decreased in Supernormal [HR, 0.729 (95% CI, 0.539–0.986)] vascular aging individuals, comparing with normal vascular aging subjects. The associations were independent of the Framingham risk score. Early vascular aging individuals also showed an elevated risk of total mortality [HR, 2.614 (95% CI, 1.302–5.249)]. Further, the associations of vascular aging categories with cardiovascular risk were much stronger in females than in males. Vascular aging categories with different cutoff levels expressed as percentiles (10th, 20th, and 25th) of Δ-age showed similar associations with cardiovascular risk.

**Conclusions:**

In conclusion, the vascular aging categories could identify people with different levels of cardiovascular risk in the Chinese population, particularly in women.

## Introduction

Cardiovascular diseases (CVD) pose a major threat to public health. According to the results of the Global Burden of Disease study, prevalent cases of total CVD events reached 523 million in 2019 (1), making CVD the leading cause of disease burden worldwide. China has become the epicenter of the CVD epidemic, where CVD has affected 330 million people and has been the cause of 40% of deaths in the Chinese population by 2019 (2). There are a variety of cardiovascular risk factors, and vascular aging (VA) or stiffening is an indispensable one.

Vascular aging is a pathophysiological process accompanying aging, characterized by arteriosclerosis, increased central pulse pressure, and endothelial dysfunction (3). Various studies have proposed distinct means of measuring the status of VA. A commonly used one was Framingham heart age, which used several conventional risk factors to estimate the patients’ cardiovascular risk and translated it to heart age (4). Besides, multiple studies incorporated results of non-invasive measurement of atherosclerosis into the existing risk models, including carotid intima-media thickness (CIMT), coronary arterial calcification (CAC), and pulse wave velocity (PWV) (5–7). In 2019, two novel concepts of VA status have been proposed by Stephane Laurent et al. (8). Early Vascular Aging (EVA) and Supernormal Vascular Aging (SUPERNOVA) are two extremes of VA distribution, representing exceptionally high or low arterial stiffness for their age and sex. EVA individuals are more liable to develop arterial stiffness and cardiovascular disease at a younger age, while SUPERNOVA individuals may still have elastic blood vessels when they are old, which is protective against CVD. The median field between EVA and SUPERNOVA is called normal vascular aging (Normal VA), where most of the population belongs to. An advantage of VA groups over other methods is that it uses both PWV and other classical cardiovascular risk factors to estimate the level of arterial stiffness, and takes into account the role of PWV other than age and blood pressure (BP) (9). Previous studies showed that VA groups implicated people at different risks of CVD and could improve risk prediction of cardiovascular events in European cohorts (9). VA groups had the potential of screening high-risk people and guiding clinical treatment. Nevertheless, the prevalence of VA phenotypes or their predictive power has not been studied in the East Asian population.

There were significant between-population differences in terms of the major type of cardiovascular event associated with arterial stiffness. Stroke is the most common cardiovascular event in East Asians, instead of ischemic heart disease (2). The slope of the association between blood pressure and stroke was steeper among Asians than Caucasians (10). Several factors such as genes and lifestyles might contribute to different associations. And increased cardiovascular risk occurred in people with lower BMI in Asian than Europeans (11, 12). Unhealthy lifestyle habits, including smoking and drinking, tended to be more common among Caucasians than Asians (13, 14). These important factors were included in our model of vascular age, which might have substantial influence on the association between vascular aging categories and cardiovascular events. For these reasons, the vascular aging categories performed well in CVD prediction in the western countries may perform differently in Chinese population. Thus, the effectiveness of EVA and SUPERNOVA in CVD prediction of East Asians and Chinese individuals require further investigation and validation.

In our study, we aimed to confirm the association of the new VA categories, EVA and SUPERNOVA with incident cardiovascular disease, CVD mortality, and all-cause mortality in a community population in Shanghai, China. Besides, we compared the associations of VA with CVD events between men and women and in categories defined by different cut-off values.

## Materials and methods

### Study population and design

**FIGURE 1:**
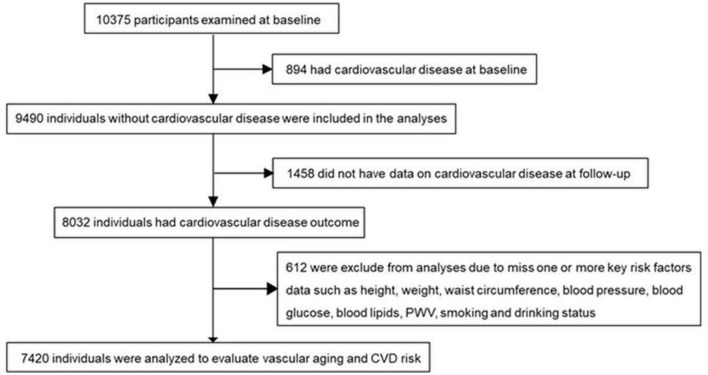
Participants flow diagram. CVD, cardiovascular disease; PWV, pulse wave velocity.

The study participants aging 40 years or older were from a cohort followed up for a median of 4.5 years in Jiading district, Shanghai (15, 16). The recruitment and baseline evaluation were completed in 2010 and the follow-up examination was conducted in 2014. As shown in Figure 1, among 10,375 participants examined at baseline, we excluded those who had CVD (*n* = 894), missing data on CVD at follow-up (*n* = 1,458), or without data on one or more key risk factors in calculating vascular age, including height, weight, waist circumstance, BP, blood glucose, blood lipids, PWV, smoking, and drinking status (*n* = 612). Finally, a total of 7,420 individuals were included for analysis. A detailed description of the population was in Supplementary Data.

The study protocol conformed to the ethical guidelines of the 1975 Declaration of Helsinki and was approved by the Institutional Review Board of Ruijin Hospital, Shanghai Jiao Tong University School of Medicine. Written informed consent was obtained from every participant. The procedures followed were in accordance with institutional guidelines.

### Clinical and laboratory measurements at baseline

A standard questionnaire was administrated to each participant by trained personnel to collect information about medical history, lifestyle, and medication use. All the measurements were performed according to a standard protocol and the details of the measurements and blood sampling protocol were described previously (17, 18).

Current smoking or drinking was defined as smoking cigarettes or consuming any kind of alcohol regularly in the past 6 months, respectively. Body height, weight, and waist circumstance were measured by trained physicians. BMI was calculated as body weight divided by squared height (kg/m^2^). Systolic blood pressure (SBP) and diastolic blood pressure (DBP) were measured three times on the non-dominant arm in a seated position after resting with an automated electronic device (OMRON Model HEM-752 FUZZY). The arm circumferences were measured to determine the cuff size before each measurement. The participants were required to avoid tea, coffee, alcohol, smoking, and exercise for at least 30 min, and take a quiet rest for at least 5 min before every measurement. The average value of the three readings was used for analysis. All participants underwent an oral glucose tolerant test (OGTT) after over 10-h fasting and blood samples were collected at 0 and 2 h during the test. Fasting blood glucose (FBG) and 2-h post-loading blood glucose (2h-PBG) were measured within 2 h after blood sample collection. Glycated hemoglobin (HbA1c), total cholesterol, triglycerides, low-density lipoprotein cholesterol (LDL-c), and high-density lipoprotein cholesterol (HDL-c) were measured using automated analyzers (Modular Analytics P800 and Modular E170). Data types and definitions of covariates involved in the study were listed in Supplementary Table 1.

Branchial-ankle pulse wave velocity (baPWV) was measured by a fully automatic arteriosclerosis diagnosis device (Colin VP-1000, Model BP203RPE II, form PWV/ABI, Japan) after at least 10-mins’ rest. Pulse waves were obtained simultaneously with suitable cuffs placed on the upper sides of bilateral arms and ankles. The distance from bilateral upper arms to ankles was corrected for its difference in time delay when obtaining the baPWV. The greater value of bilateral baPWV was adopted for analysis.

To eliminate the influence of measurement errors on our results, we performed data cleaning in this study. We eliminated implausible data that violated logic and dealt with data out of measurement range by checking raw records of anthropometric data or re-examining the blood sample for biochemical data and deleted those which were still out of range.

### Vascular age calculation and definitions of vascular aging categories

We used a multivariable linear regression model which included baPWV, multiple classical CVD risk factors, and treatment to estimate vascular age (8, 9). Variables were selected by the backward stepwise selection approach. The final model included the following variables [sex, CVD family history, SBP, BMI, waist circumstance, FBG, 2h-PBG, HbA1c, Total cholesterol, triglyceride, HDL-c, smoking and drinking status, baPWV, and BP-lowering treatment (9)]. Most of them and baPWV showed a non-linear relationship with age, so we used log-transformation, Generalized Additive Models (GAM), or 2nd-degree polynomials to improve the prediction performance of the model, evaluated by the R^2^ and the Akaike Information Criterion. In the final model, all of the continuous variables included were log-transformed, and the calculation formula and detailed description of parameters for the equation are shown in Supplementary Table 2. The model of vascular age was further validated by 10-fold cross-validation in our cohort. The R square was 0.341 (*P* < 0.001), indicating that the model fitted well. The Pearson-R correlation coefficient of chronological age and vascular age was 0.592 and the intraclass correlation coefficient was 0.518 (*P* < 0.001), showing that the two ages were moderately correlated.

Δ-age was calculated as chronological minus vascular age. To define EVA and SUPERNOVA, we calculated the 10^th^ and 90^th^ percentiles of Δ-age. The EVA individuals have extremely high vascular age and the Δ-age was lower than 10^th^ percentiles, while the SUPERNOVA subjects had rather low vascular age and their Δ-age was over 90^th^ percentiles.

### Primary outcome ascertainment

The primary outcome of the current analysis was the composite of incident fatal and non-fatal cardiovascular events, including cardiovascular mortality, myocardial infarction, and stroke. We collected the disease and mortality information on vital status from the local municipal health authorities. Two members of the outcome adjudication committee independently checked each outcome event and discrepancies were adjudicated by other committee members.

### Statistics

Baseline characteristics of the participants were described as mean ± standard deviation (SD) for normally distributed continuous variables, median (interquartile range) for skew-distributed continuous variables, and frequency (percentage) for categorical variables. Differences among participants in EVA, Normal VA, and SUPERNOVA groups were examined by unpaired *t*-test (for normally distributed continuous variables), Wilcoxon rank sum test (for skew-distributed continuous variables), and chi-square test (for categorical variables). Atherosclerotic Cardiovascular Disease (ASCVD) risk score, calculated by the Pooled Cohort Equations in the 2013 ACC/AHA Guideline on the Assessment of Cardiovascular Risk (19), was also compared among three VA categories.

We used the Normal VA group as the reference in the analysis because EVA and SUPERNOVA were two extreme phenotypes. To detect the association between VA categories and outcomes, cox proportional hazards models were used to calculate hazard ratios (HRs) and 95% confidence intervals (95%CI). In model 1, age and sex were the adjusted covariates; model 2 additionally adjusted for the 2008 General Framingham risk score (FRS) (4); model 3 included the ASCVD risk score based on model 1. The Schoenfeld residual analysis of variables in the Cox models demonstrated that all the variables satisfied the PH assumption, except for sex. So, we further performed the analysis in sex subgroups.

**FIGURE 2:**
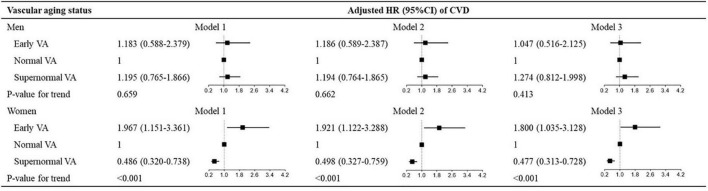
Hazard ratios for VA categories for CVD in sex subgroups. Model 1: adjusted for age and sex; model 2: model 1 + Framingham risk score; model 3: model 1 + ASCVD risk score. VA, vascular aging; CVD, cardiovascular disease; ASCVD, atherosclerotic cardiovascular disease; HR, hazard ratio; CI, confidence interval.

**FIGURE 3:**
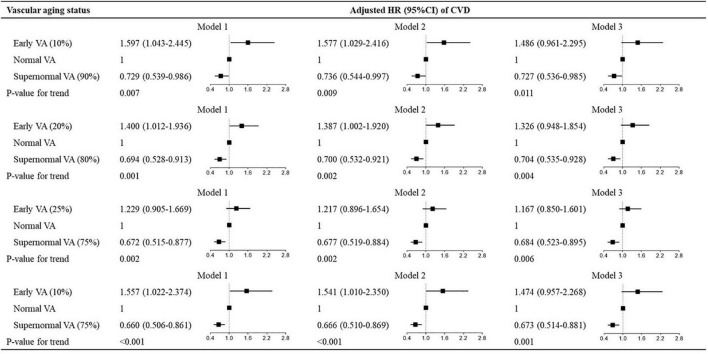
Hazard ratios for different cutoff values of vascular aging categories for CVD. Hazard ratios for vascular aging categories for CVD. The cutoff values for the three vascular aging categories are, respectively, defined by 10% and 90%, 20% and 80%, 25% and 75%, and 10% and 75% percentiles of Δ-age. Model 1: adjusted for age and sex; model 2: model 1 + Framingham risk score; model 3: model 1 + ASCVD risk score. VA, vascular aging; CVD, cardiovascular disease; ASCVD, atherosclerotic cardiovascular disease; HR, hazard ratio; CI, confidence interval.

In the next analysis, we stratified participants into subgroups according to sex (men vs. women) and cutoff values of Δ-age (10% and 90% vs. 20% and 80% vs. 25% and 75% vs. 10% and 75% percentiles of Δ-age) and examined the associations between VA categories and the outcomes in each sex and cutoff-point subgroup in the total cohort. The results were shown in Figures 2, 3. We also divided participants according to BMI and waist circumstance and the results were shown in Supplementary Tables 7, 8.

In the additional analyses, an association between Δ-age as a continuous variable rather than VA categories and outcomes was estimated in Cox survival analysis, adjusted for the same covariates (Supplementary Table 3). We also compared the predictive effects of ASCVD risk score, Δ-age, and the combined model with the basic model including age and sex using Harrel’s C-statistic (Supplementary Table 4). To investigate the possible mechanisms behind sex differences in vascular aging, some of the menstrual and gestational characteristics of female participants in EVA, Normal VA, and SUPERNOVA were compared by chi-square test (Supplementary Table 5). To further eliminate the influence of measurement errors on our results, we performed a sensitivity analysis (Supplementary Table 6). We log-transformed the continuous variables in the vascular age calculation model to let them fit normal distributions and eliminated the data out of mean ± 3 SD according to the PauTa (3σ) criteria (20). After this, 222 participants with outliers were excluded and 7,198 participants were included in the sensitivity analysis.

A two-sided *P*-value < 0.05 was considered statistically significant. All analyses were performed using SAS software, version 9.4 (SAS Institute, Cary, NC, United States).

## Results

Based on the definition of VA categories mentioned before, we recognized participants of the three groups. Baseline socioeconomic and biochemical characteristics of them are listed in Table 1. With similar vascular age, EVA individuals were 12 years younger and SUPERNOVA individuals were 14 years older than Normal VA individuals. A majority of cardiovascular risk factors showed statistically significant differences among the three VA categories. The baPWV and systolic and diastolic BP were the highest in EVA group and the lowest in SUPERNOVA group, respectively. The blood glucose profile, including FBG, 2h-PBG, and HbA1c, showed lower levels in the SUPERNOVA group than in the EVA group but did not have a significant difference. Notably, the mean ASCVD risk score of individuals in the SUPERNOVA category was three times higher than that of people in the Normal VA group, while EVA individuals had the lowest ASCVD risk score.

**TABLE 1:** Baseline clinical characteristics of three vascular aging categories individuals.

Variables	Early VA	Normal VA	Supernormal VA	*P*-value
Number of participants	742	5936	742	–
Chronological age (year)	46.68 ± 5.57	57.59 ± 7.56	72.88 ± 6.07	< 0.001
Vascular age (year)	58.82 ± 5.95	57.26 ± 5.50	58.50 ± 5.06	< 0.001
Δ-age (year)	−12.14 ± 2.73	0.33 ± 4.97	14.38 ± 3.47	< 0.001
Female sex (%)	55.12%	63.93%	57.28%	< 0.001
Current smoker (%)	22.24%	20.75%	21.97%	0.518
Current drinker (%)	11.46%	10.56%	11.32%	0.653
CVD family history (%)	10.65%	15.75%	7.82%	< 0.001
BMI (kg/m^2^)	25.00 ± 3.30	25.18 ± 3.21	24.72 ± 3.36	< 0.001
Waist circumference (cm)	82.55 ± 9.25	82.51 ± 8.79	81.56 ± 9.31	0.021
baPWV (m/s)	16.37 ± 4.10	15.49 ± 3.33	16.04 ± 3.08	< 0.001
Systolic BP (mm Hg)	143.19 ± 21.38	140.50 ± 19.85	141.73 ± 19.19	0.001
Diastolic BP (mm Hg)	88.99 ± 11.42	82.87 ± 9.94	77.29 ± 9.71	< 0.001
FBG (mmol/L)	5.62 ± 1.91	5.54 ± 1.47	5.51 ± 1.41	0.327
2h-PBG (mmol/L)	8.30 ± 4.83	8.18 ± 4.26	7.90 ± 3.88	0.161
HbA1c (%)	5.88 ± 1.22	5.82 ± 0.90	5.82 ± 0.89	0.266
Triglycerides (mg/dL)	117.7 (81.4,169.9)	122.1 (86.7,174.3)	111.5 (81.4,154.0)	< 0.001
Total Cholesterol (mg/dL)	199.0 (176.1,225.5)	204.6 (181.1,230.3)	200.6 (176.1,223.2)	< 0.001
LDL-c (mg/dL)	116.6 (97.7,140.9)	122.0 (101.9,144.0)	118.1 (95.4,138.2)	< 0.001
HDL-c (mg/dL)	51.0 (42.5,59.8)	49.8 (42.1,59.8)	51.4 (42.9,61.4)	0.060
BP-lowering treatment (%)	1.89%	1.60%	1.89%	0.742
ASCVD risk score (%)	1.43 (0.75,3.22)	3.98 (2.15,7.35)	13.08 (8.33,24.00)	< 0.001

VA, vascular aging; baPWV, brachial-ankle pulse wave velocity; BMI, body mass index; BP, blood pressure; CVD, cardiovascular disease; LDL-c, low-density lipoprotein cholesterol; HDL-c, high-density lipoprotein cholesterol; FBG, fasting blood glucose; 2h-PBG, OGTT 2h post-load blood glucose; HbA1c, glycated hemoglobin; ASCVD, atherosclerotic cardiovascular disease.

During a median follow-up period of 4.5 years, 403 participants developed incident cardiovascular disease. Cardiovascular mortality was documented in 40 individuals. As shown in Table 2, after adjusting for age and sex, EVA individuals had 59.7% [HR, 1.597 (95% CI, 1.043–2.445)] higher risk of developing cardiovascular events than Normal VA individuals. By contrast, SUPERNOVA individuals had 27.1% [HR, 0.729 (95% CI, 0.539–0.986)] lower risk of suffering cardiovascular events than Normal VA individuals. The associations between VA categories and incident CVD events maintained significant after further adjusting for FRS. But the significance disappeared in the EVA group after adjusting for the ASCVD risk score. We did not find any significant association between VA categories and stroke, cardiac events, or CVD mortality, while EVA participants showed elevated all-cause mortality [HR, 2.614 (95% CI, 1.302–5.249)]. The association among EVA, SUPERNOVA, and cardiovascular risk remained significant after outlier elimination (Supplementary Table 6).

**TABLE 2:** Hazard ratios for vascular aging categories for total mortality, CVD, stroke, and cardiac events.

Outcomes	VA categories	HR (95%CI)		
		Model 1	Model 2	Model 3
Cardiovascular events	Early VA	1.597 (1.043–2.445)	1.577 (1.029–2.416)	1.486 (0.961–2.295)
	Normal VA	1	1	1
	Supernormal VA	0.729 (0.539–0.986)	0.736 (0.544–0.997)	0.727 (0.536–0.985)
Stroke	Early VA	1.331 (0.832–2.130)	1.310 (0.818–2.097)	1.255 (0.777–2.027)
	Normal VA	1	1	1
	Supernormal VA	0.791 (0.563–1.113)	0.802 (0.570–1.129)	0.788 (0.560–1.110)
Cardiac events	Early VA	1.734 (0.347–8.662)	1.761 (0.350–8.845)	1.674 (0.324–8.646)
	Normal VA	1	1	1
	Supernormal VA	0.923 (0.273–3.122)	0.915 (0.270–3.100)	0.927 (0.273–3.142)
CVD mortality	Early VA	4.392 (0.946–20.380)	4.324 (0.927–20.177)	3.860 (0.805–18.503)
	Normal VA	1	1	1
	Supernormal VA	0.503 (0.228–1.107)	0.506 (0.229–1.116)	0.509 (0.230–1.129)
Total mortality	Early VA	2.614 (1.302–5.249)	2.629 (1.307–5.289)	2.343 (1.150–4.775)
	Normal VA	1	1	1
	Supernormal VA	1.245 (0.857–1.809)	1.241 (0.853–1.805)	1.268 (0.869–1.850)

Model 1: adjusted for age and sex; model 2: model 1 + Framingham risk score; model 3: model 1 + ASCVD risk score.

VA, vascular aging; CVD, cardiovascular disease; ASCVD, atherosclerotic cardiovascular disease; HR, hazard ratio; CI, confidence interval.

Figure 2 shows the multivariate-adjusted HRs for CVD events in association with VA categories in sex subgroups. VA categories were significantly associated with CVD risk in women across three models (all *P* < 0.001), but the association disappeared in men. For the other outcomes, VA categories had a stronger association with stroke, instead of cardiac events, CVD mortality, or total mortality in women than in men (Supplementary Tables 9, 10).

To explore the predictive values of VA categories with different cutoff points, we investigated the association between VA categories and CVD risk in subgroups stratified by different cutoff values. As shown in Figure 3, the multivariate-adjusted HRs for CVD varied in VA categories with different cutoff values expressed by percentiles of Δ-age (10th, 20th, 25th, 10th for EVA and 90th, 80th, 75th, 75th for SUPERNOVA). The associations between VA categories and CVD events showed modest differences among subgroups (all P for trend < 0.05). In women, the associations between VA categories of different cutoff values and CVD risk were also similar (Supplementary Table 11).

The association between VA and cardiovascular events remained significant in the overweight/obese individuals but was not significant in the individuals with normal weight. The association also was borderline significant in the central obese subjects, but not significant in the subjects with normal waist circumstances (Supplementary Tables 7, 8). However, the P for interaction for both subgroup results was not significant (*P* > 0.05).

In the additional analyses, Δ-age was significantly associated with the incidence of CVD, adjusted for age and sex [HR, 0.962 (95% CI, 0.944–0.981)]. One-year difference between chronological and vascular age (Δ-age) was associated with 3.8% less CVD risk (Supplementary Table 3). We further compared the predicting value of the basic model, ASCVD risk score, and Δ-age (Supplementary Table 4). ASCVD risk score significantly improved the predicting value of age and sex model by 0.4% [ΔC-statistics, 0.004 (95% CI, 0.001–0.008), *P* = 0.012]. Δ-age and the combined model including both Δ-age and ASCVD score, respectively, increased the predictive effect of the age and sex model by 1.0% [ΔC-statistics, 0.010 (95% CI, 0.001–0.018), *P* = 0.024] and 1.1% [ΔC-statistics, 0.011 (95% CI, 0.003–0.020), *P* = 0.011], which indicated that combining Δ-age and ASCVD risk score had a significant effect of improving the predictive value for CVD in age and sex model.

## Discussion

In this community prospective cohort study, participants in the EVA group and SUPERNOVA group exhibited significantly higher or lower risk of CVD events, respectively, compared with those in the Normal VA group. The associations between VA categories and cardiovascular risk were independent of the Framingham risk score. The clinical relevance of VA categories was more significant in females than in males.

To the best of our knowledge, it was the first prospective study to show that EVA and SUPERNOVA were associated with different risks of incident CVD events in the Chinese population. EVA syndrome is a concept first described in 2008, which stands for premature arterial stiffness and increased PWV in younger patients (21, 22). On the contrary, the SUPERNOVA phenotype has not been proposed until 2019, which is defined as individuals whose cfPWV is extremely lower than their age and sex predict (8). We used a model which included baPWV and multiple classical CVD risk factors to estimate vascular age and defined VA categories based on the variation between chronological and vascular age. In this way, we successfully recognized participants in EVA, Normal VA, and SUPERNOVA, who accounted for 10%, 80%, and 10%, respectively, of our study population.

The clinical relevance of EVA and SUPERNOVA has been rarely explored by prospective studies. A European study performed by Bruno et al. in a group of elderly individuals (over 60 years old) followed-up by 6.6 years found that the HR of CVD in the SUPERNOVA group was 0.59 (95% CI, 0.41–0.85) and HR of CVD in EVA group was 2.70 (95% CI, 1.55–4.70). The results remained significant after adjustment for age and sex or ASCVD risk score (9). In accordance with previous findings, we demonstrated that EVA and SUPERNOVA both were significantly associated with the risk of incident cardiovascular events, even after adjusting for age and sex. The EVA individuals had a 59.7% higher risk of developing cardiovascular events. And the risk of SUPERNOVA individuals was 27.1% lower than that of Normal VA individuals. Moreover, VA categories were independent predictors of CVD events beyond the Framingham risk score in our study. Although EVA was partly associated with ASCVD risk score in the total cohort, both EVA and SUPERNOVA were independent of the two classical CVD predictors in females. The EVA individuals also had a higher all-cause mortality rate compared to Normal VA individuals, which was consistent with the results of the aforementioned study.

There were some differences between the results of the study performed by Bruno et al. and our study. First, DBP and heart rate were not included in our vascular age model. They did not promote the fitting effect significantly. The association between DBP and arterial stiffness has been reported in multiple studies. A quantitative meta-analysis supported that there was no significant association between baseline DBP and progression of arterial stiffness, while the association was stronger between SBP and arterial stiffness (23). The probable reason was that SBP was more strongly dependent on the pulsatile component of BP than DBP. Besides, the major target organ of DBP was the heart, instead of peripheral arteries, and the fluctuation range of DBP was smaller than SBP. Moreover, the associations between SBP and cardiovascular events were different between the European and Chinese populations (10). Thus, the effect of DBP on vascular aging might be covered by SBP in our cohort. The association between heart rate and arterial stiffness has been in debate for a long time. It was reported that although heart rate was related to PWV, further adjustment on sex, age, and BP reduced its residual influence on PWV which was very small (24). To sum up, the effects of DBP and heart rate on PWV might be covered by SBP or other risk factors in the model based on our Chinese cohort, and further analysis was needed to confirm the findings.

Second, the mean (SD) of delta-age in our study was higher than that in the Bruno et al. study, probably because the chronological age of individuals in our cohort ranged from 40 to 80 years, while the age of participants in the Bruno et al. study was over 60 years. The large range of age allowed us to observe individuals with extremely higher or lower vascular age.

Third, the association between vascular aging categories and CVD events was stronger in the Bruno et al. study than in our study. The possible reasons were as follows. First, the participants in our study were younger and were followed-up for a relatively shorter period, so fewer cardiovascular events occurred in our study and the associations showed less effect. Second, there were more young women in our EVA group, who had protective factors like high estrogen level and their risk of developing CVD events could be much lower compared to the aged women in the EVA group of Bruno et al. At last, the EVA participants in the European study were from developed countries and under relatively good health management, for instance, their blood pressure was controlled well, whereas they still had an extremely high level of arterial stiffness. By contrast, a majority of the participants in our study did not receive optimal healthcare and had poor control of their blood pressure. So, the extreme vascular aging phenotype in the study of Bruno et al. might be more “extreme” due to the difference in healthcare levels.

It is noteworthy that most of the baseline characteristics of EVA and SUPERNOVA individuals were similar, except for their chronological age. The 10-year ASCVD risk of EVA group participants was less than 20% of that in the SUPERNOVA group estimated by Pooled Cohort Equations of ASCVD risk, since chronological age is the most important risk factor in predicting 10-year cardiovascular event risk in the equations (19). Thus, the ASCVD risk score might overestimate the cardiovascular risk of EVA and underestimate that of SUPERNOVA individuals. Compared with the ASCVD risk score, the VA categories performed better in selecting high-risk young people and low-risk old people who had similar cardiometabolic profiles, which may provide guidance for clinics and avoid deficient or excessive medical treatment (25). In the additional analyses, we found that the combination of ASCVD risk score and Δ-age significantly improved the predicting effect of the age and sex model for CVD events, which further suggested the clinical application prospects of VA categories.

Furthermore, in the subgroup analysis, we found that the association between VA categories and CVD events, especially stroke was significant in females, but disappeared in males. Arterial stiffness and CVD risk increase linearly with age in men, but women experience a curvilinear aging trend, with the time of menopause as a turning point (26). This is possibly due to perimenopausal estrogen loss, which is hazardous to the cardiovascular system of females (27). Moreover, there are various cardiometabolic risk factors that majorly exist in women, such as polycystic ovarian syndrome, contraception, gestational complications, and autoimmune disorders (28). Accordingly, the VA process of females could show more diversity than that of males, influenced by multiple risk elements, and predict different levels of CVD risk. Some pathogenetic mechanisms of arterial stiffening might be responsible for this phenomenon, including extracellular matrix alterations, oxidative stress, inflammation, and Renin-Angiotensin II-Aldosterone System signaling (29). There is evidence of sex and aging-related differences in gene expression and functions of angiotensin II type 2 receptor in the kidney and brain of mice (30). The meticulous sex-specific vascular aging mechanisms require further research.

The cardiovascular risk of women was significantly higher in the EVA group and lower in the SUPERNOVA group than in the Normal VA group, despite the fact that SUPERNOVA females were much older and have lost vascular protection from estrogen, which was meant to be cardio-protective in EVA females (27). The association remained significant after adjustment for FRS or ASCVD score. The effect of estrogen may be attenuated by other risk factors. We compared the prevalence of some female-specific characteristics in the three VA groups and the result was shown in Supplementary Table 5. We found that gestational hypertension, gestational diabetes mellitus, and recurrent miscarriages were more prevalent in EVA women than in SUPERNOVA women, which might relate to vascular aging. Therefore, it is possible for some elder females to retain elastic arteries if they have healthy cardiometabolic profiles or favorable medical history. VA categories were more efficient in distinguishing high-risk women from low-risk ones when compared with men.

Various upper and lower thresholds of Normal VA were tested for predicting CVD in our study. The definitions of EVA and SUPERNOVA were arbitrary and no consensus has been reached for the lower and upper thresholds of EVA and SUPERNOVA (8). Laurent supported that the use of the 10^th^ and 90^th^ or 25^th^ and 75^th^ percentiles of cfPWV was appropriate to define the threshold values (31). We decided to define EVA and SUPERNOVA with 10^th^ and 90^th^ percentiles because it was the most commonly used threshold in the previous studies (9, 32). Epidemiological studies comparing the predictive values of EVA and SUPERNOVA according to various lower and upper thresholds are lacking (8). So, we chose the cutoff values used in previous studies to define vascular aging categories to compare the predictive values of EVA and SUPERNOVA in our cohort (21). Our study demonstrated that using these specific percentiles to define vascular aging categories would not change the association substantially. Future researches are needed to specifically describe the distribution of cardiovascular risk changing with Δ-age and determine the cutoff values for VA categories according to study purposes.

The combined or interactive effect of vascular aging with BMI or waist circumstance on cardiovascular risk was seldom investigated in previous studies. We found a stronger association between vascular aging and cardiovascular risk in those with high BMI and waist circumstances. It was possible that BMI and waist circumstance were included in the vascular age model, so the difference in associations was more evident in those with abnormal BMI or waist circumstances or those with metabolic diseases.

This study has several strengths. To the best of our knowledge, it was the first study to explore the association of VA categories with incident cardiovascular disease in a large Chinese community cohort. What’s more, it was a prospective study and we used hard CVD events and mortality as our outcome indicators. In addition, we performed population stratification and verified the association in sex and cutoff value subgroups.

Our study also has notable limitations. First, our cohort only recruited individuals over 40 years old. However, CVD events mainly occur in middle-aged and old people, so the inclusion of young adults may not significantly influence our results. Second, we did not test our model in other independent validation cohorts. Validation of this concept in other East Asian populations was essential to expand its applicability.

In conclusion, in a Chinese prospective cohort, participants in the EVA group and SUPERNOVA group exhibited significantly higher or lower risk of CVD events, respectively, compared with those in the Normal VA group. In addition, VA categories were more efficient in screening high- and low-risk people in females than in males. Our results emphasized that VA categories may be potential tools to identify middle-aged and elder Chinese people at different risks of developing CVD.

## Data availability statement

The original contributions presented in this study are included in the article/Supplementary material, further inquiries can be directed to the corresponding author/s.

## Ethics statement

The studies involving human participants were reviewed and approved by the Institutional Review Board of Ruijin Hospital, Shanghai Jiao Tong University School of Medicine. The patients/participants provided their written informed consent to participate in this study.

## Author contributions

GN, WW, YB, and ZZ conceived and designed the study. QC performed data analyses and drafted the manuscript. ML, TW, YC, MD, DZ, YX, MX, and JL recruited patients and collected data. All authors contributed to the article and approved the submitted version.
