# Supplementary material for: Association of Early and Supernormal Vascular Aging categories with cardiovascular disease in the Chinese population
Source: Front Cardiovasc Med. 2022 Aug 11;9:895792. doi: 10.3389/fcvm.2022.895792 (PMC9403083; doi:10.3389/fcvm.2022.895792)
Supplement: Supplementary file 1 [file Data_Sheet_1.pdf]

frontiers

# *Supplementary Material*

## 1 Supplementary Data

### 1.1 Study population

This is a large prospective cohort of participants aged⩾40 years from Jiading district, Shanghai, China. As shown in the flowchart in Figure 1, the study population (n=7420) for the present analysis was selected from the 10375 individuals in the whole cohort according to the following exclusion criteria: overt CV disease; missing records of cardiovascular diseases at follow-up; missing values among the following covariates: height; weight; waist circumstances; systolic and diastolic BP; fasting blood glucose; 2h post-load blood glucose; glycated hemoglobin; total, LDL and HDL cholesterol; triglycerides; PWV; smoking and drinking status.

### 1.2 Vascular age calculation

We used a multivariable linear regression model which included baPWV, multiple classical CVD risk factors and treatment to estimate vascular age. We included multiple classical CVD risk factors in our initial model (sex, CVD family history, SBP, DBP, heart rate, BMI, waist circumstance, FBG, 2h-PBG, HbA1c, total cholesterol, triglyceride, LDL-C, HDL-C, smoking and drinking status, baPWV and BP-lowering treatment) and selected them by backward stepwise selection approach. DBP, heart rate and LDL-C were excluded in our final model, because they did not promote the fitting effect significantly.

### 1.3 Definition of EVA and SUPERNOVA categories

EVA and SUPERNOVA categories were defined by 10th and 90th percentile of Δ-age, which was calculated as chronological minus vascular age. The clinical characteristics of EVA, normal VA and SUPERNOVA individuals were compared by effect size calculation. Atherosclerotic Vascular Disease (ASCVD) risk score, calculated by the Pooled Cohort Equations in the 2013 ACC/AHA Guideline on the Assessment of Cardiovascular Risk, was also compared among three VA categories. It represented the 10-year cardiovascular risk of participants with the same race and sex. A higher ASCVD score stood for higher risk of CVD events.

### 1.4 Association of VA categories with CVD events and mortality

A Cox survival analysis was used to test the association between VA categories and outcomes. Model 1 adjusted for age and sex. Model 2 further adjusted for the 2008 general Framingham Risk Score. Model 3 additionally adjusted for ASCVD risk score beyond age and sex.

The primary outcome of our analyses was the composite of incident fatal or nonfatal CVD events including myocardial infarction, stroke and cardiovascular death. Myocardial infarction was defined by symptoms of myocardial ischemia, characteristic changes in value of troponin T and creatine-kinase-MB isoform, changes in electrocardiogram results or a combination of them. Stroke was defined as a fixed neurologic deficit at least 24 hours due to a presumed vascular cause. Deaths were collected from vital registries of the National Disease Surveillance Point System and National Health Insurance System. Two members of the outcome adjudication committee independently checked

each outcome event and discrepancies were adjudicated by other committee members. All committee members were unaware of the baseline risk factors of participants.

### 1.5 Analyses in sex and cut-off values subgroups

Association between VA categories and outcome was verified in sex subgroups (males, females) and cut-off values subgroups (10% and 90% vs. 20% and 80% vs. 25% and 75% vs. 10% and 75% percentiles of Δ-age). The adjusted factors in model 1,2,3 were the same as those in the main analysis.

## 2 Supplementary Figures and Tables

### 2.1 Supplementary Tables

**Table S1. Data types and definitions of covariates involved in the study**

| Covariate | Data type during analysis | Categorical variable definition or continuous variable unit |
|---|---|---|
| Education | Categorical variable | Less education: less than high school (<9 years)<br>Higher education: high school or further (≥9 years) |
| Physical activity | Categorical variable | Physical inactivity: leisure-time physical activity of 0 to <600 MET-min/week<br>Ideal physical activity: leisure-time physical activity of ≥600 MET-min/week (the equivalent of 150 minutes each week of brisk walking or 75 minutes per week of running) |
| Alcohol consumption | Categorical variable | Current alcohol drinker: consuming alcohol at least once a week for at least 6 months<br>Not Current alcohol drinker: never consumed alcohol or had not consumed alcohol regularly |
| Smoking | Categorical variable | Current smoker: smoking every day or almost every day, with at least 7 cigarettes per week for at least 6 months<br>Not Current smoker: never smoked or had not smoked cigarettes regularly (at least 7 cigarettes per week for at least 6 months in a lifetime) |
| Family history of CVD | Categorical variable | Yes: At least one of the first-degree relatives was diagnosed as CVD<br>No: No one among first-degree relatives was diagnosed as CVD |

| | | |
|---|---|---|
| Treatment for hypertension | Categorical variable | Yes: Taking at least one antihypertensive medication<br>No: Never took any antihypertensive medication |
| Sex | Categorical variable | / |
| Systolic blood pressure | Continuous variable | mm Hg |
| Diastolic blood pressure | Continuous variable | mm Hg |
| BMI | Continuous variable | $kg/m^2$ |
| Waist circumstance | Continuous variable | cm |
| FBG | Continuous variable | mmol/L |
| 2h-PBG | Continuous variable | mmol/L |
| HbA1c | Continuous variable | % |
| Total cholesterol | Continuous variable | mg/dl |
| LDL-c | Continuous variable | mg/dl |
| HDL-c | Continuous variable | mg/dl |
| Triglycerides | Continuous variable | mg/dl |

BMI, body mass index; HDL, high-density lipoprotein cholesterol; LDL-c, low-density lipoprotein cholesterol; MET, metabolic equivalent; CVD, cardiovascular disease; FBG, fasting blood glucose; 2h-PBG, OGTT 2h post-load blood glucose; HbA1c, glycated hemoglobin.

**Table S2. Parameters of equation for vascular age calculation**

| Parameters, units | Coefficient | Data type |
|---|---|---|
| log (BaPWV), cm/s | 0.398 | Continuous variable |
| Sex (1=Man, 0=Woman) | -0.032 | Categorical variable |
| log (treated SBP), mmHg | 0.050 | Continuous variable |
| log (untreated SBP), mmHg | 0.045 | Continuous variable |
| log (BMI), $kg/m^2$ | -0.156 | Continuous variable |
| log (Waist circumstance), cm | 0.226 | Continuous variable |
| log (FBG), mmol/L | -0.102 | Continuous variable |
| log (2h-PBG), mmol/L | 0.024 | Continuous variable |
| log (HbA1c), % | 0.106 | Continuous variable |
| log (Triglyceride), mmol/L | -0.017 | Continuous variable |
| log (HDL-c), mmol/L | 0.058 | Continuous variable |
| log (Total cholesterol), mmol/L | 0.026 | Continuous variable |
| Current smoking (1=Yes, 0=No) | -0.040 | Categorical variable |
| Current drinking (1=Yes, 0=No) | -0.011 | Categorical variable |
| CVD family history (1=Yes, 0=No) | -0.035 | Categorical variable |

BaPWV, branchial-ankle pulse wave velocity; SBP, systolic blood pressure; BMI, body mass index; HDL, high-density lipoprotein cholesterol; CVD, cardiovascular disease; FBG, fasting blood glucose; 2h-PBG, OGTT 2h post-load blood glucose; HbA1c, glycated hemoglobin. The unit of blood lipids variables were converted to SI units (The International System of Units) when modeling.

**Table S3. Hazard ratio for vascular aging categories and for Δ-age as continuous variable for cardiovascular events, coronary events, stroke and total mortality**

| | | HR for VA categories (95%CI) | HR for Δ-age (95%CI) |
|---|---|---|---|
| Cardiovascular events | EVA | 1.597 (1.043-2.445) | 0.962 (0.944-0.981) |
| | Normal VA | 1 | |
| | SUPERNOVA | 0.729 (0.539-0.986) | |
| | Age | 1.075 (1.061-1.088) | 1.088 (1.072-1.105) |
| | Sex | 1.006 (0.823-1.229) | 1.009 (0.826-1.233) |
| Cardiac events | EVA | 1.734 (0.347-8.662) | 0.977 (0.903-1.056) |
| | Normal VA | 1 | |
| | SUPERNOVA | 0.923 (0.273-3.122) | |
| | Age | 1.064 (1.011-1.121) | 1.073 (1.008-1.142) |
| | Sex | 0.386 (0.169-0.883) | 0.384 (0.168-0.878) |
| Stroke | EVA | 1.331 (0.832-2.130) | 0.967 (0.947-0.988) |
| | Normal VA | 1 | |
| | SUPERNOVA | 0.791 (0.563-1.113) | |
| | Age | 1.062 (1.047-1.077) | 1.076 (1.058-1.094) |
| | Sex | 1.112 (0.890-1.388) | 1.117 (0.894-1.395) |
| Total mortality | EVA | 2.614 (1.302-5.249) | 0.991 (0.965-1.017) |
| | Normal VA | 1 | |
| | SUPERNOVA | 1.245 (0.857-1.809) | |
| | Age | 1.115 (1.094-1.136) | 1.122 (1.098-1.147) |
| | Sex | 0.644 (0.484-0.855) | 0.641 (0.483-0.852) |

EVA, early vascular aging; Normal VA, normal vascular aging; SUPERNOVA, supernormal vascular aging; HR, hazard ratio; CI, confidence interval.

**Table S4. Comparison of effects in different predicting models for cardiovascular events**

| Model | C-statistic (95%CI) | ΔC-statistic (95%CI) | P for ΔC |
|---|---|---|---|
| Age + Sex | 0.673(0.646,0.700) | - | - |
| Age + Sex +ASCVD score | 0.677(0.650,0.704) | 0.004(0.001,0.008) | 0.012 |
| Age + Sex + Δ-age | 0.682(0.655,0.709) | 0.010(0.001,0.018) | 0.024 |
| Age + Sex + Δ-age + ASCVD score | 0.684(0.657,0.711) | 0.011(0.003,0.020) | 0.011 |

ASCVD, atherosclerotic cardiovascular disease; CI, confidence interval. The C-statistic of each model was compared with that of "Age + Sex" model to calculate the ΔC-statistic.

**Table S5. The prevalence of sex-specific characteristics in EVA, Normal VA and SUPERNOVA women**

| Risk factors | EVA | Normal VA | SUPERNOVA | P for trend |
|---|---|---|---|---|
| Menopause | 24.21% | 80.45% | 100.00% | <0.001 |
| Premature ovarian failure (menopause before the age of 45 years) | 4.06% | 5.48% | 10.29% | <0.001 |
| Gestational hypertension | 5.38% | 1.87% | 0.94% | <0.001 |
| Gestational Diabetes Mellitus | 0.49% | 0.08% | 0.00% | 0.044 |
| Recurrent miscarriages (>1 time) | 23.47% | 30.54% | 19.53% | <0.001 |

EVA, early vascular aging; Normal VA, normal vascular aging; SUPERNOVA, supernormal vascular aging.

**Table S6. Hazard ratios for vascular aging categories for CVD events and total mortality after elimination of outliers**

| Outcomes | VA categories | HR (95%CI) | | |
|---|---|---|---|---|
| | | Model 1 | Model 2 | Model 3 |
| Cardiovascular events | EVA | 1.608 (1.028-2.516) | 1.596 (1.019-2.499) | 1.530 (0.968-2.418) |
| | Normal VA | 1 | 1 | 1 |
| | SUPERNOVA | 0.650 (0.437-0.894) | 0.654 (0.476-0.899) | 0.647 (0.470-0.890) |
| Total mortality | EVA | 2.864 (1.355-6.050) | 2.863 (1.353-6.059) | 2.499 (1.164-5.364) |
| | Normal VA | 1 | 1 | 1 |
| | SUPERNOVA | 1.144 (0.771-1.698) | 1.144 (0.770-1.699) | 1.160 (0.778-1.730) |

EVA, early vascular aging; Normal VA, normal vascular aging; SUPERNOVA, supernormal vascular aging; HR, hazard ratio; CI, confidence ratio; CVD, cardiovascular diseases.

**Table S7. Hazard ratios for vascular aging categories for CVD events in BMI subgroups**

| | | VA categories | HR (95%CI) | | |
|---|---|---|---|---|---|
| | | | Model 1 | Model 2 | Model 3 |
| Women | BMI<25 | EVA | 1.476 (0.674-3.231) | 1.480 (0.673-3.254) | 1.403 (0.625-3.151) |
| | | Normal VA | 1 | 1 | 1 |
| | | SUPERNOVA | 0.607 (0.331-1.113) | 0.605 (0.329-1.115) | 0.601 (0.327-1.105) |
| | BMI≥25 | EVA | 2.655 (1.272-5.541) | 2.575 (1.232-5.383) | 2.441 (1.143-5.214) |
| | | Normal VA | 1 | 1 | 1 |
| | | SUPERNOVA | 0.406 (0.226-0.730) | 0.418 (0.231-0.754) | 0.397 (0.220-0.718) |
| Men | BMI<25 | EVA | 1.494 (0.553-4.035) | 1.526 (0.562-4.143) | 1.242 (0.449-3.432) |
| | | Normal VA | 1 | 1 | 1 |
| | | SUPERNOVA | 1.128 (0.598-2.126) | 1.117 (0.592-2.110) | 1.292 (0.670-2.491) |
| | BMI≥25 | EVA | 0.971 (0.363-2.597) | 0.970 (0.362-2.594) | 0.898 (0.333-2.422) |
| | | Normal VA | 1 | 1 | 1 |
| | | SUPERNOVA | 1.254 (0.670-2.347) | 1.254 (0.670-2.347) | 1.275 (0.679-2.393) |

EVA, early vascular aging; Normal VA, normal vascular aging; SUPERNOVA, supernormal vascular aging; HR, hazard ratio; CI, confidence ratio; BMI, body mass index; CVD, cardiovascular diseases.

**Table S8. Hazard ratios for vascular aging categories for CVD events in central obesity subgroups**

| | | VA categories | HR (95%CI) | | |
|---|---|---|---|---|---|
| | | | Model 1 | Model 2 | Model 3 |
| Women | Without central obesity | EVA | 1.861 (0.800-4.328) | 1.858 (0.798-4.327) | 1.562 (0.652-3.739) |
| | | Normal VA | 1 | 1 | 1 |
| | | SUPERNOVA | 0.549 (0.281-1.074) | 0.551 (0.280-1.082) | 0.538 (0.273-1.060) |
| | | EVA | 2.060 (1.029-4.123) | 1.983 (0.987-3.981) | 2.002 (0.980-4.092) |

| | | | | | |
|---|---|---|---|---|---|
| | With central obesity | Normal VA | 1 | 1 | 1 |
| | | SUPERNOVA | 0.476 (0.276-0.821) | 0.488 (0.282-0.845) | 0.472 (0.272-0.817) |
| Men | Without central obesity | EVA | 1.219 (0.465-3.195) | 1.260 (0.479-3.317) | 1.064 (0.399-2.836) |
| | | Normal VA | 1 | 1 | 1 |
| | | SUPERNOVA | 0.927 (0.541-1.589) | 0.917 (0.535-1.573) | 1.020 (0.585-1.776) |
| | With central obesity | EVA | 0.957 (0.344-2.662) | 0.952 (0.342-2.650) | 0.905 (0.322-2.542) |
| | | Normal VA | 1 | 1 | 1 |
| | | SUPERNOVA | 2.146 (0.979-4.707) | 2.132 (0.972-4.677) | 2.146 (0.972-4.737) |

EVA, early vascular aging; Normal VA, normal vascular aging; SUPERNOVA, supernormal vascular aging; HR, hazard ratio; CI, confidence ratio; CVD, cardiovascular diseases.

**Table S9. Hazard ratios for vascular aging categories for stroke, cardiac events, CVD mortality and total mortality in men**

| Outcomes | VA categories | HR (95%CI) | | |
|---|---|---|---|---|
| | | Model 1 | Model 2 | Model 3 |
| Stroke | EVA | 0.757 (0.315-1.823) | 0.764 (0.317-1.840) | 0.681 (0.281-1.652) |
| | Normal VA | 1 | 1 | 1 |
| | SUPERNOVA | 1.476 (0.886-2.459) | 1.473 (0.884-2.454) | 1.562 (0.933-2.616) |
| Cardiac events | EVA | 2.029 (0.353-11.661) | 2.087 (0.360-12.093) | 2.018 (0.337-12.064) |
| | Normal VA | 1 | 1 | 1 |
| | SUPERNOVA | 2.013 (0.442-9.171) | 1.989 (0.436-9.068) | 2.018 (0.437-9.325) |
| CVD mortality | EVA | 6.700 (1.252-35.854) | 6.177 (1.153-33.092) | 5.471 (0.973-30.743) |
| | Normal VA | 1 | 1 | 1 |
| | SUPERNOVA | 0.423 (0.133-1.340) | 0.426 (0.134-1.351) | 0.473 (0.147-1.525) |
| Total mortality | EVA | 2.408 (0.886-6.545) | 2.226 (0.833-5.948) | 2.294 (0.856-6.151) |
| | Normal VA | 1 | 1 | 1 |
| | SUPERNOVA | 1.024 (0.612-1.713) | 1.073 (0.649-1.774) | 1.066 (0.644-1.763) |

EVA, early vascular aging; Normal VA, normal vascular aging; SUPERNOVA, supernormal vascular aging; HR, hazard ratio; CI, confidence ratio; CVD, cardiovascular diseases.

**Table S10. Hazard ratios for vascular aging categories for stroke, cardiac events, CVD mortality and total mortality in women**

| Outcomes | VA categories | HR (95%CI) | | |
|---|---|---|---|---|
| | | Model 1 | Model 2 | Model 3 |
| Stroke | EVA | 1.812 (1.039-3.161) | 1.754 (1.004-3.062) | 1.663 (0.937-2.953) |
| | Normal VA | 1 | 1 | 1 |
| | SUPERNOVA | 0.488 (0.305-0.782) | 0.506 (0.315-0.814) | 0.477 (0.297-0.768) |
| Cardiac events | EVA | ------ | ------ | ------- |
| | Normal VA | 1 | 1 | 1 |
| | SUPERNOVA | 0.209 (0.020-2.177) | 0.208 (0.020-2.199) | 0.210 (0.020-2.174) |
| CVD mortality | EVA | ------ | ------ | ------- |
| | Normal VA | 1 | 1 | 1 |
| | SUPERNOVA | 0.511 (0.164-1.596) | 0.461 (0.143-1.484) | 0.504 (0.160-1.581) |
| Total mortality | EVA | 3.061 (1.140-8.220) | 2.952 (1.096-7.951) | 2.308 (0.828-6.436) |
| | Normal VA | 1 | 1 | 1 |

| | | | |
|---|---|---|---|
| SUPERNOVA | 1.480 (0.842-2.601) | 1.534 (0.886-2.717) | 1.548 (0.865-2.768) |

EVA, early vascular aging; Normal VA, normal vascular aging; SUPERNOVA, supernormal vascular aging; HR, hazard ratio; CI, confidence ratio; CVD, cardiovascular diseases. The association could not be analyzed in subgroups with no events, and the HR (95%CI) was showed by "------".

**Table S11. Hazard ratios for different cutoff values of vascular aging categories for CVD events in women**

| VA categories | HR (95%CI) | | |
|---|---|---|---|
| | Model 1 | Model 2 | Model 3 |
| EVA (10%) | 1.967 (1.151-3.361) | 1.921 (1.122-3.288) | 1.800 (1.035-3.128) |
| Normal VA | 1 | 1 | 1 |
| SUPERNOVA (90%) | 0.486 (0.320-0.738) | 0.498 (0.327-0.759) | 0.477 (0.313-0.728) |
| P for trend | <0.001 | <0.001 | <0.001 |
| EVA (20%) | 1.608 (1.073-2.410) | 1.575 (1.049-2.364) | 1.537 (1.005-2.349) |
| Normal VA | 1 | 1 | 1 |
| SUPERNOVA (80%) | 0.538 (0.375-0.773) | 0.548 (0.381-0.789) | 0.543 (0.377-0.781) |
| P for trend | <0.001 | <0.001 | <0.001 |
| EVA (25%) | 1.506 (1.030-2.202) | 1.477 (1.009-2.164) | 1.456 (0.978-2.170) |
| Normal VA | 1 | 1 | 1 |
| SUPERNOVA (75%) | 0.561 (0.396-0.797) | 0.572 (0.402-0.814) | 0.567 (0.399-0.807) |
| P for trend | <0.001 | <0.001 | <0.001 |
| EVA (10%) | 1.832 (1.078-3.112) | 1.794 (1.055-3.052) | 1.749 (1.012-3.023) |
| Normal VA | 1 | 1 | 1 |
| SUPERNOVA (75%) | 0.550 (0.388-0.779) | 0.561 (0.395-0.798) | 0.557 (0.392-0.793) |
| P for trend | <0.001 | <0.001 | <0.001 |

EVA, early vascular aging; Normal VA, normal vascular aging; SUPERNOVA, supernormal vascular aging; HR, hazard ratio; CI, confidence ratio; CVD, cardiovascular diseases.
